# An Innovative Multi-Omics Approach Reveals the Interactions Between Honeybees and Their Environment

**DOI:** 10.3390/ani15182660

**Published:** 2025-09-11

**Authors:** Cecilia Rudelli, Elisa Bellei, Giulia Andreani, Gloria Isani

**Affiliations:** 1 Department of Veterinary Medical Sciences, University of Bologna, 40064 Bologna, Italy; giulia.andreani2@unibo.it (G.A.); gloria.isani@unibo.it (G.I.); 2 Proteomic Laboratory, Department of Surgery, Medicine, Dentistry and Morphological Sciences, University of Modena and Reggio Emilia, 41125 Modena, Italy; elisa.bellei@unimore.it

**Keywords:** proteomics, health assessment, honeybees, vitellogenin, essential elements

## Abstract

Although traditional methods of evaluating colony health provide a general overview, they are often subjective and imprecise. Therefore, a more integrative approach is needed. The aim of this study is to use a multi-omics strategy—combining proteomic and metallomic approaches—to evaluate the interactions between honeybees and their environment. Two apiaries were selected for the study: apiary A, which is located in an extensive agricultural landscape, and apiary M, which is located in a less anthropized environment. The distribution of honeybee soluble proteins and small biomolecules was analyzed using size-exclusion chromatography. These profiles varied significantly between sampling times and apiaries. During spring, a reduced high molecular mass protein peak and an elevated very low molecular mass molecules peak were observed in honeybees from apiary A. Hemolymph analysis corroborated these findings, showing a depletion of key nutritional proteins, such as vitellogenin and apolipophorin in this apiary during the same season. In addition to protein distribution, we studied also the speciation of zinc and copper using chromatographic and atomic absorption techniques. Our results indicate site and season-dependent variation in metal binding profiles, with evidence for the presence of metallothionein-like proteins capable of binding both copper and zinc. This study highlights the urgent need for integrated, multi-method research to understand the complex dynamics of honeybee health and emphasizes the fundamental role of agricultural practices in ensuring the health of honeybees.

## Simple Summary

It is becoming increasingly important to evaluate honeybee health in more precise and objective way. This study applied a combined approach of protein and metal analysis to gain a better understanding of how honeybees interact with their environment. The analysis revealed significant variations in the types and quantities of proteins and small molecules present in honeybees from different locations and seasons. Honeybees’ handling of metals such as zinc and copper also varied depending on the location and time of year. These results emphasize the significant influence that environmental conditions and agricultural practices can have on bee health.

## 1. Introduction

Although traditional methods of evaluating colony health provide a broad overview, they are often subjective and imprecise. Recently, molecular diagnostic tools, such as honey environmental DNA [1] or the Bee Health Card, have been proposed. This latter method is an interesting laboratory tool developed to assess biotic and abiotic stressors using the MALDI (Matrix-Assisted Laser Desorption Ionization) Bee Typing ©, which analyses the hemolymph peptidome of honeybees [2]. Additionally, hemolymph proteins have demonstrated great potential as biomarkers for assessing the nutritional and health status of honeybees. Like plasma in vertebrates, hemolymph can be considered an open window onto the honeybee’s physiological machinery. Additionally, the honeybee’s circulatory system is open, allowing direct contact between the hemolymph and tissues. In two recent papers we have proposed and tested a panel of hemolymph proteins in field condition as a useful tool to assess the health and nutritional status of honeybees at the colony level [3,4]. These proteins, namely vitellogenin, apolipophorins, transferrin, and hexamerin 70a were found to be significantly correlated with each other as well as with the traditional measures of colony performance such as adult population and brood size, and pollen and honey reserves.

Now it is time to go one step further. Building on this foundation, it is time to adopt a more integrative approach that connects honeybee nutrition and health to environmental factors. Honeybees are widely recognized as a model for the One Health concept and serve as excellent bioindicators of environmental contamination. Honeybees accumulate environmental trace elements and are therefore used as bioindicators [5,6,7]. Honeybees collect these elements both directly, through the airborne particulate matter captured by their cuticular hairs during flight [8], and indirectly, by drinking water and foraging on environmental sources such as nectar, pollen, resins, and honeydew, which have a characteristic elemental profile. Consequently, trace elements have been quantified in honeybees from different environments around the globe [7,9,10]. However, studies on element speciation, i.e., the distribution of a specific element among defined chemical species in a biological system, are rare in honeybees [11]. Coupling of size exclusion chromatography (SEC) with elemental detection using sensitive techniques, such as atomic absorption spectrometry (AAS) or inductively coupled plasma-mass spectrometry (ICP-MS), is a well-established approach for screening the presence of metal–biomolecule complexes in biological samples and has been used previously also in Hymenoptera [12,13].

This study aims to use a multi-omics strategy—combining proteomic and metallomic approaches—to evaluate the interactions between honeybees and their environment. This approach offers a valuable tool for the detection of colony stress across varying environments, thereby advancing field-based health assessments. Here, we selected two of the four apiaries studied by Isani et al. 2023 [3] and sampled honeybees in four critical periods throughout the year from April to November, in order to cover the entire production season and also include the winter honeybees, which are vital for the survival of the colony. The two apiaries were owned by the same beekeeper who managed them in the same way, but they were located in sites with different levels of anthropic impact and, consequently, botanical biodiversity: the first apiary was surrounded by an extensive agricultural landscape characterized mostly by wheat and sunflower cultivation, while the second apiary was located on a hill surrounded by vineyards, wild flora and a richer biodiversity. The most pronounced differences between colonies were observed in May and in November, which are two critical months for honeybee colonies. The former is when disparities in floral abundance across the environments surrounding the apiaries are most pronounced, and the latter is when honeybees prepare for the overwintering phase. We hypothesize that variations in environmental conditions may influence honeybee protein and zinc and copper profiles.

## 2. Materials and Methods

### 2.1. Experimental Design, Colony Management, and Hemolymph Sampling

The first apiary (named apiary A) was located in Argelato, an extensive agricultural landscape (44°38′40.6″ N 11°19′49.6″ E) characterized by wheat and sunflowers, while the second apiary (named apiary M) was located in Musiano, a less anthropized environment, characterized by the presence of vineyards and a higher abundance of wild flora (44°22′51.0″ N 11°20′00.3″ E). The same professional beekeeper owned both apiaries, and none of the colonies showed any clinical signs of disease. To treat varroosis, the colonies received Apivar strips (Véto-Pharma, Palaiseau, France) in August and November (when the samplings were already finished). Three representative colonies were chosen from each apiary, ensuring they had similar anamnesis (*n* = 3). All the colonies were uniform in terms of bee and brood numbers and had adequate honey and pollen stores. The samplings were performed in 2022.

Honeybees from each of three hives in each apiary were sampled between the last brood frame and the stores to minimize age variability during four seasonal periods: April, May, July, and November. Nurse honeybees were selected for sampling, as they are mainly present over the brood. Only nurse bees were collected because they have higher concentrations of hemolymph proteins, particularly vitellogenin, than forager bees [14]. From each hive and at each sampling time, 30 honeybees were collected for hemolymph analysis, while 10 were collected for Size Exclusion Chromatography (SEC) analysis. The honeybees were transported refrigerated to the Department of Veterinary Medical Sciences, where 1–2 microlitres of transparent, uncontaminated hemolymph were immediately collected from each bee using a glass microcapillary (Blaubrand^®^ BRANDT GmbH, Wertheim, Germany), which was inserted between the fourth and fifth tergite. At least one microlitre of hemolymph was obtained from each honeybee. Other honeybees were stored at −80 °C for subsequent analyses.

### 2.2. Size Exclusion Chromatography (SEC) of Honeybee Extracts

Whole honeybees were used to obtain soluble protein extracts for analysis. For each apiary and at each sampling time, honeybees from the three colonies were pooled and one gram was homogenised in 5 volumes (*w*/*v*) of Tris-HCl 20 mM and 10 mM mercaptoethanol, pH 8.6, using an Ultraturrax homogeniser (IKA, Staufen, Germany). The resulting homogenate was then centrifuged at 24,000 g for 30 min at 4 °C in a Beckman Coulter TJ-25 centrifuge (Beckman Coulter, Brea, CA, USA); the resulting supernatant was centrifuged again at the same speed for further 30 min at 4 °C. The supernatant was then isolated and fractionated using size exclusion chromatography (SEC). For each extract, a volume of 0.8 mL of the supernatant was applied to a Sephadex G-75 column (0.9 × 90 cm). The column was calibrated using a commercial kit (MWGF70-1KT, Sigma-Aldrich, St. Louis, MO, USA). The elution buffer consisted of Tris-HCl 20 mM containing 5 mM 2-mercaptoethanol. Fractions of 1.5 mL were collected. Proteins were analysed by measuring the absorbance at 280 nm using a DeNovix DS-11 Spectrophotometer (Wilmington, DE, USA).

### 2.3. Protein Separation Using SDS-PAGE

Proteins were separated using 1D-SDS-PAGE (one dimensional Sodium Dodecyl Sulfate Poly-Acrylamide Gel Electrophoresis). Hemolymph samples were diluted 1:40 and chromatographic fractions were analysed undiluted. For each sample, three microliters were loaded on 4–12% Bis-Tris polyacrylamide gels (NuPage/Thermo Fisher Scientific, Waltham, MA, USA) and electrophoresis was carried out in an Xcell SureLock Mini-Cell with 2-(N-morpholino) propanesulfonic acid buffer (MOPS; NuPage/Thermo Fisher Scientific, Waltham, MA, USA) at pH 7.3 containing sodium dodecyl sulfate (SDS). Each gel was also loaded with standard proteins of known molecular mass (SeeBlue™ Pre-stained Protein Standard, Thermo Fisher Scientific, Waltham, MA, USA). The electrophoresis system was connected to a power supply (Power Pack Basic—Bio-Rad, Hercules, CA, USA) with a constant voltage of 200 V for 40 min. The gels were stained with Coomassie G250. After staining, each gel was digitalised using ChemiDocMP (BioRad, Hercules, CA, USA), and pherograms were obtained using ImageLab 5.2.1 software (BioRad, Hercules, CA, USA). One µg of protein, obtained from a solution containing 1 µg/µL of lactate dehydrogenase (LDH), (Sigma-Aldrich/Merck KGaA, Darmstadt, Germany) was added to each sample as an internal standard.

### 2.4. Protein Identification by Mass Spectrometry

After performing SDS-PAGE on the hemolymph from the honeybees sampled in May from Apiary A, the bands of interest were manually excised from the gel for protein identification. The bands were destained using acetonitrile, and the proteins were then reduced with 10 mM dithiothreitol and alkylated with 55 mM iodoacetamide. The proteins were digested with trypsin (Promega, Madison, WI, USA) at 37 °C, and the peptides obtained were concentrated in a vacuum dryer (Savant Speed vac, model DNA 110- PROGITEC S.r.l., Sabaudia (LT), Italy). The samples were analysed using an UHPLC-MS QExactive™ (Thermo Fisher Scientific, Reinach, Switzerland) system, composed of UHPLC 3000 Ultimate System coupled to an ESI-QExactive™ Hybrid Quadrupole-Orbitrap™ mass spectrometer (LC-ESI-QO-MS/MS System), as previously described [15]. Protein identification was obtained using the MASCOT search engine (version 2.7.0, www.matrixscience.com accessed on 8 March 2024) against the UniProt database for peptide sequences, and C-RAP for contaminants (accessed on 8 March 2024), setting the following restriction parameters: taxonomy, all entries (a broader taxonomy was selected for identification based on sequence homology, as the honeybee hemolymph protein database is not yet well annotated), trypsin as a proteolytic enzyme (max one missed cleavage), peptide mass tolerance ± 10 ppm and fragment mass tolerance ± 0.04 Da, carbamidomethyl (C) (fixed modifications), deamidated (NQ) and oxidation (M) (variable modifications). The significant threshold was set to obtain a false discovery rate < 1%. Only proteins with the highest score hits among the MASCOT search results and identified with at least two or more significant sequences were selected.

### 2.5. Metal Analysis Using Atomic Absorption Spectrometry (AAS)

Size exclusion chromatography was performed as described in 2.2. Copper and zinc were analysed in fractions obtained from size exclusion chromatography by aspirating the liquid directly into the flame of an atomic absorption spectrophotometer (AAnalyst 100, Perkin Elmer, Waltham, MA, USA). The accuracy of the method was evaluated by analysing an international standard (European Certified Reference Material, ERM^®^—BB422 fish muscle). The concentrations obtained using this method fell within the certified uncertainty interval provided by ERM, corresponding to a 95% confidence level. The detection limits 0.04 µg/mL for zinc (Zn) and 0.01 µg/mL for copper (Cu). Concentrations of trace elements were reported as μg/mL.

### 2.6. Statistical Analysis

Regarding hemolymph proteins, the differences between groups at the same sampling time were determined using the Kruskal–Wallis test. A *p*-value < 0.05 was considered statistically significant. The statistical analyses were performed using R 4.2.1 (R Foundation for Statistical Computing; Vienna, Austria; https://www.R-project.org/, accessed on 30 January 2025).

## 3. Results and Discussion

The health of honeybees is closely linked to environmental conditions, with changes in resource availability, climatic fluctuations, and anthropogenic pressures playing a crucial role in their physiological status [16]. In this context, soluble protein profiles and metal-binding patterns of *A. mellifera* provide valuable insights into the impact of environmental quality on honeybee biochemistry. This research integrates chromatographic, electrophoretic, and spectrometric data to explore the influence of seasonal and spatial variation on the biochemical composition of honeybees.

### 3.1. The Quality of the Environment Affects Honeybee Proteins

**Figure 1:**
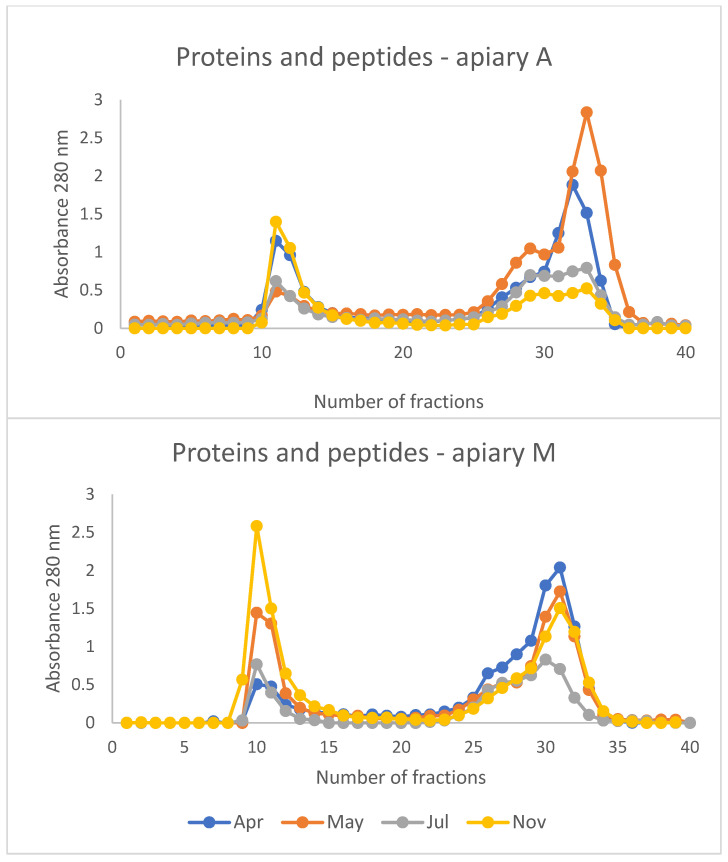
Chromatographic patterns after size exclusion chromatography (SEC) of extracts of honeybees sampled from apiaries A and M and in different periods of the year. Proteins, peptides and metabolites were measured at 280 nm.

Figure 1 shows the profiles of molecules which absorb at 280 nm in the SEC fractions. Two major peaks are present in all the obtained chromatograms. The first peak is present between fractions 10 and 14, with maximum absorbance at 280 nm measured in fraction 11 in all the chromatograms obtained. This peak contains high molecular mass (HMM) proteins between >75 and 40 kilodaltons (kDa). These are the soluble proteins found in the honeybee body and are involved in cell function, metabolism, and regulation.

The second peak is present between fractions 30 and 36, with the maximum absorbance occurring between fractions 30 and 33. This peak contains very low molecular mass (VLMM) molecules, such as free amino acids and other metabolites that absorb at 280 nm. Some chromatograms also show a peak/shoulder between fractions 26 and 30, which contains small proteins and peptides with molecular mass in the range of 6–4 kDa (low molecular mass (LMM) molecules), and is particularly evident in the chromatographic profile of the honeybee extract from apiary A in May.

The highest peak of HMM proteins was present in both apiaries in November, while the highest peak of VLMM molecules was found in the apiary A in May and in the apiary M in April. Low HMM and VLMM peaks were present in both apiaries in July.

In May, the extracts of honeybees from apiary A had the lowest peak of HMM proteins. At the same time, the peaks in fractions 26–30 and 30–36 reached their maximum intensity, indicating a higher concentration of LMM and VLMM molecules. This may be due, at least in part, to a higher concentration of peptides and free amino acids rather than proteins, as reflected by the low HMM proteins peak.

To verify this hypothesis, the proteins present in fraction 11 of the honeybee extracts obtained from samples collected in May and November from both apiaries were separated using SDS-PAGE. The results are reported in Supplementary Figure S1. Comparing the electrophoretic profiles reveals qualitative and quantitative differences. The amount of protein in honeybees from apiary A in May is lower, and different protein bands are present, particularly in the range between 60 kDa and 28 kDa. Conversely, the profiles of the extracts from both apiaries are similar in November, but the sample from honeybees in apiary M had a higher protein concentration, in line with the chromatographic profiles.

**Figure 2:**
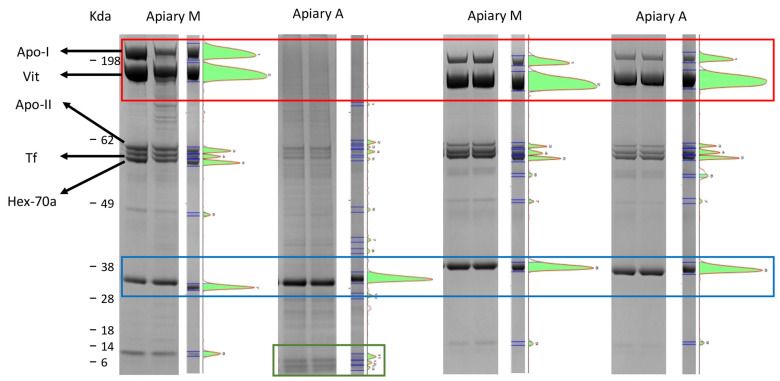
SDS-PAGE (Sodium Dodecyl Sulfate Poly-Acrylamide Gel Electrophoresis) gels and pherograms of hemolymph proteins of honeybees of apiary A and apiary M in May (on the left) and in November (on the right). Apo-I is apolipophorin I, Apo-II is apolipophorin II, Vit is vitellogenin, Tf is transferrin, Hex-70a is hexamerin 70a. The red box highlights the vitellogenin and apolipophorin bands which were absent in honeybees from apiary A in May. The blue box shows the internal standard of quantity (1 μg). The green box shows the bands that were cut out for protein mass identification. The molecular masses of the standard proteins are reported on the left.

This finding aligns with the electrophoretic profile of hemolymph proteins which reveals an absence of vitellogenin and apolipophorin I, which are HMM proteins, as well as significantly lower concentrations of apolipophorin II, transferrin and hexamerin 70a in honeybees from apiary A in May (*p* < 0.05) (Figure 2). This suggests protein degradation and the presence of low molecular mass proteins or peptides, as evidenced by the presence of tiny additional bands of low molecular mass proteins in the gel of honeybees from apiary A and in the soluble proteins extracted from whole honeybees (Figure 1). Conversely, similar hemolymph protein profiles were observed in honeybees from both apiaries at the end of the production season in November, before the winter period (Figure 2).

We chose to analyze also the hemolymph due to the open circulatory system in honeybees, which puts the hemolymph in direct contact with all body districts, and because hemolymph is an ideal sample for protein identification using mass spectrometry [3]. To verify the presence of proteolytic products of the aforementioned proteins, we cut out the bands corresponding to molecular masses of ≤14 kDa and analyzed them using mass spectrometry to discover which proteins were present. The proteins identified are listed in Supplementary Table S1. Alongside low molecular mass proteins present in honeybee hemolymph, including various odorant-binding proteins (OBP), which is consistent with previously published data [3], we also identified fragments of vitellogenin, which could be the result of proteolytic cleavage of the protein. No fragments of the other proteins were identified. It is important to note that the electrophoretic protocol used in this study has a detection threshold of approximately 10 kDa; therefore, the presence of peptides below this molecular mass cannot be evidenced and cannot be definitively excluded.

Honeybee vitellogenin, produced by the fat body and released into the hemolymph is a multifarious high molecular mass protein that performs various interconnected functions. It is involved in energy storage and distribution, which contributes to the nutritional state of the honeybee and plays a role in maintaining metabolic balance [17,18,19]. Hemolymph vitellogenin can indeed be considered as a circulating reservoir of amino acids and other molecules or ions, such as zinc, which cells can use to sustain physiological processes. Vitellogenin can be broken down to provide metabolic substrates when nutrient demands increase, for example, during the winter or in times of stress, or when there is a shortage of pollen and nectar in the environment. Therefore, a low concentration of circulating vitellogenin may indicate a stressful condition. Nutritional and environmental stressors such as heat, starvation and chemical exposure can interfere with vitellogenin metabolism. This can result in the degradation or reduced synthesis of the protein through the interaction of various regulatory pathways [20].

We could ask why such a situation occurred in apiary A in May. Excluding pest infestation, diseases, and inadequate hive management—the apiary was checked weekly and the same professional beekeeper owned apiary M too—the most relevant factors that may have caused stressful conditions are probably limited food diversity and foraging opportunities. This apiary is located in an agricultural area that relies on monoculture crops, and the wild plants that bloom in March and April, do not provide sufficient nectar and pollen sources afterwards.

Conversely, it could be argued that the honeybees from apiary M, which is located in a less anthropized environment characterized by a variety of wild flora, were in a better nutritional state than those from apiary A. At the start of wintering phase in November, the honeybees in apiary M had a higher peak of HMM proteins, indicating a higher amount of total soluble proteins in their body (Figure 1 and Supplementary Figure S1). Despite having similar electrophoretic profiles, the honeybees from apiary M had a higher protein content in November. Therefore, it can be hypothesised that honeybees from apiary M were in a better trophic state throughout the sampling period from May to November.

It is known that environmental exploitation and agriculture have an impact on the wild flora, consequently affecting honeybee foraging activity [21]. Accordingly, Smart et al. 2019 [22] reported that honeybees from apiaries located in less cultivated areas have higher levels of vitellogenin transcripts. The data reported in our research provide evidence that this is also true at the protein level. Winter bees generally have higher concentrations of hemolymph total proteins and vitellogenin than summer bees as this is an adaptation that enables them to survive the cold season [3,4,19]. Therefore, the presence of high concentrations of soluble total body and specific hemolymph proteins during the critical pre-winter period is considered as a key factor in determining whether a colony will succeed or collapse after winter [19,23].

Finally, similar profiles were observed in both apiaries in July, most likely due to the climate change and limited food resources available in both environments in this month. In Emilia-Romagna, 2022 was the hottest year since 1961, with high temperatures in July and an unusually warm spring [24]. The summer drought was severe, with a hydroclimatic deficit of -395 mm across the region, which impaired the development of wild flora. In fact, it has been reported that a dry climate could reduce nectar and pollen production [25]. Despite their ability to regulate the hive’s temperature, these climate variations can negatively impact honeybee thermoregulation and threaten colony homeostasis. Flores et al. 2018 [26] observed lower increases in hive weight in summer 2017 than in 2016, as well as a decrease in the adult bee population. This was associated with reduced flowering period by three weeks and a decreased amount of nectar and pollen available to honeybees. Variations in temperature and precipitation affect also plants, potentially ending flower blooms prematurely and reducing consequently the availability of pollen and nectar. Scarcity of food resources is problematic, because colonies facing this situation exhibit behavioural modifications, such as the earlier initiation of foraging, which is likely driven by lipid depletion [27,28]. The diversity and quality of available food resources impact also other aspects of bee physiology, including microbiota composition, and pathogen resistance. These factors in turn influence colony development, honey production, and the survival and longevity of the colony [29,30]. To mitigate this threat, beekeepers provide alternative nutritional sources to prevent colony losses and maintain honey production levels [31], although in this study the beekeeper did not supplement the colonies.

The study of metabolites present in the VLMM peaks is beyond the scope of this research. Metabolomics, the comprehensive study of metabolites within a biological system, such as a cell, tissue, or organism, is still in its infancy in honeybee research [21,32]. However, metabolomics could provide valuable insights into the complex physiological and biochemical responses of honeybees to environmental quality and food resources. The chromatographic profiles in Figure 1 show differences between apiaries and sampling times which warrant further investigation in this field in future research.

### 3.2. Zinc and Copper Speciation Is Affected by Environmental Factors

To gain a deeper understanding of the relationship between honeybees and their environment, the speciation of copper and zinc was addressed using hyphenated techniques. These techniques involve the chromatographic fractionation of metal-binding molecules followed by sensitive metal detection in the fractions using atomic absorption spectrometry.

**Figure 3:**
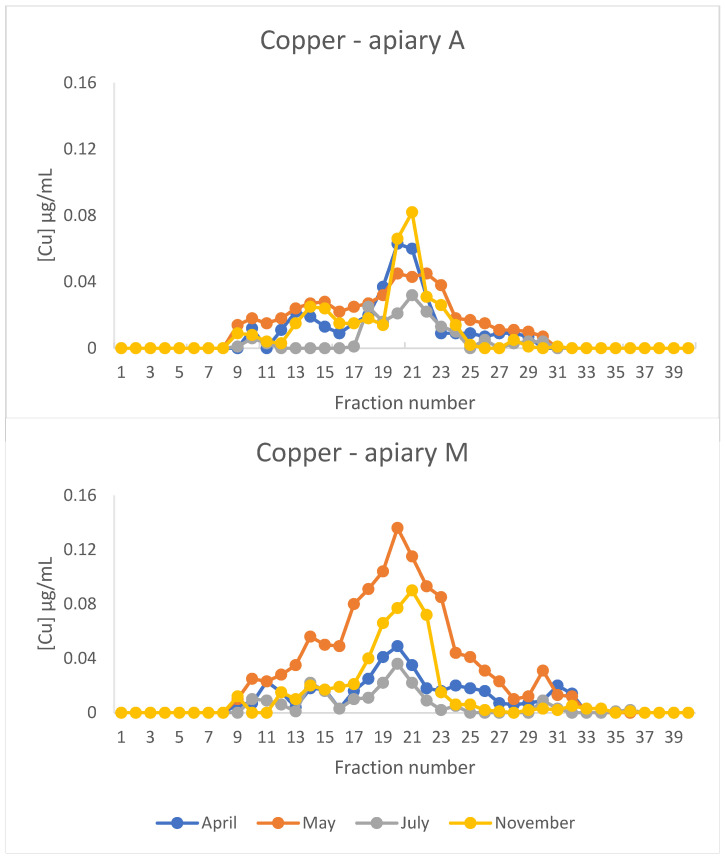
Chromatographic profiles of copper after size exclusion chromatography (SEC) of extracts of honeybees sampled from apiaries A and M in different periods of the year. The concentration is expressed in µg/mL.

Figure 3 shows the copper profiles from the cytosolic extracts of honeybees. Copper showed a major peak in fractions 18–23 in all the profiles obtained. This peak was particularly evident in the extract of honeybees sampled in May from apiary M. A similar copper-containing peak has also been reported in hornets [12]. Other minor peaks were present in fractions 9–11 and 13–16. The first peak corresponds to HMM molecules, while the peak in fractions 12–16 probably contains the superoxide dismutase, as has also been suggested in hornets [12].

**Figure 4:**
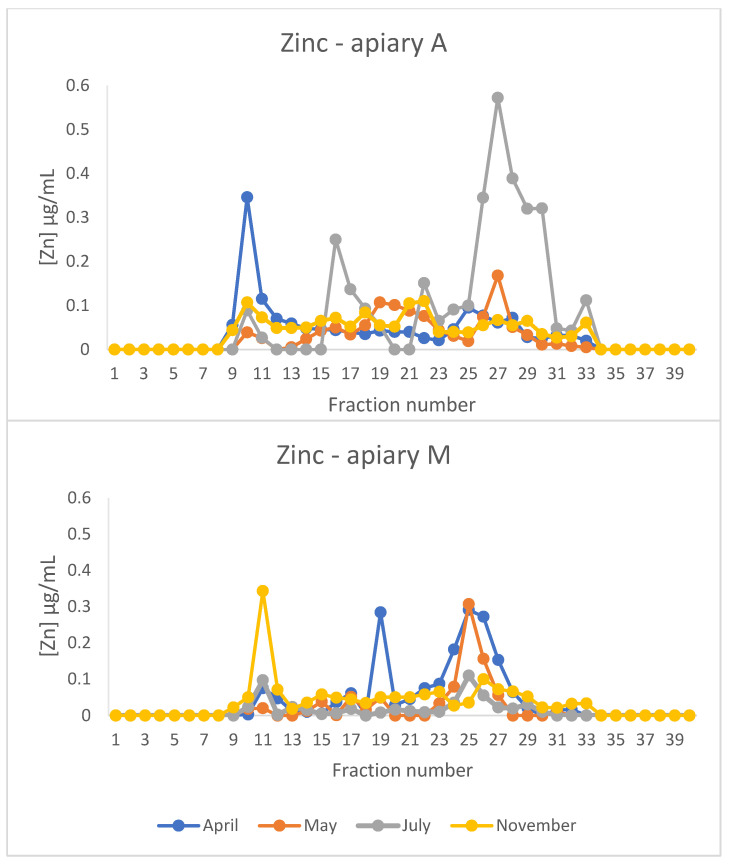
Chromatographic profiles of zinc after size exclusion chromatography (SEC) of extracts of honeybees sampled from apiaries A and M in different periods of the year. The concentration is expressed in µg/mL.

Figure 4 shows the zinc profiles from the cytosolic extracts of honeybees after gel filtration chromatography. Zinc showed different peaks depending on the sampling period and was spread throughout the chromatograms. A peak in the HMM fractions (between fractions 9 and 11) was present in all the profiles. This peak was more prominent in apiary A in April and in apiary M in November. A second evident peak is present in fractions 24–29, corresponding to LMM proteins/peptides. A third peak was present in fractions 18–22, which is superimposable with the copper peak.

The presence of a peak containing Cu and Zn is a common finding in chromatograms from vertebrate and invertebrate cytosolic extracts [33]. This peak contains metallothioneins (Mts). They are small, cysteine-rich metal-binding proteins that play a crucial role in metal homeostasis and detoxification, particularly in response to environmental exposure to heavy metals, in a wide range of animals, from invertebrates [34], including honeybees [35], to mammals [33]. Only one gene encoding a putative Mt (*AmMT*) has been identified in *A. mellifera* [35]. However, to the authors’ knowledge, Mt has never previously been isolated in honeybees. This may be the first evidence of the presence of the protein in this species.

Honeybees are considered interesting bioindicators of heavy metal contamination in the environment because they reflect the levels of contamination of soil, air, water, and plants in the areas where they live and forage [10,11,36]. Elements such as arsenic and lead are present in glyphosate-containing pesticides [37], while copper sulphate is widely used to treat vineyards and horticultural crops, even in organic farming, as it is the only accepted treatment under the European Organic Farming Regulation [38,39,40]. Of particular interest is the situation observed in May in honeybees from apiary M: the high peak of copper reflects the copper sulphate treatments of vineyards in the proximity of the apiary. In this case the induction of Mt is due to the necessity of binding the excess of this redox element to avoid the formation of reactive oxygen species and oxidative stress. Copper is toxic to honeybees. A laboratory experiment involving *A. mellifera carnica* determined an LC_50_ of 66 mg/L following 10 days of chronic oral exposure to various concentrations of CuSO_4_ [41]. However, concentrations in the range of 0.002–20 mg/L, comparable to or lower than those found in contaminated environments, did not induce malaise in honeybees. The authors therefore concluded that, in a natural setting, sources with these values of copper contamination may not adversely affect foraging honeybees [42].

Metal specificity is a critical issue regarding the biochemistry of Mts. The genome of *Drosophyla melanogaster* contains six Mt loci, designated as *MtnA* to *MtnF*. All these Mt isoforms are considered as Cu-thioneins, except for MtnF, which is thought to be a Zn-thionein [43]. Therefore, drosophyla Mts primarily act as Cu-thioneins in comparison to their mammalian counterparts, which predominantly are Zn-thioneins. The putative Mt present in fractions 18–22 can bind zinc and copper, and the predominant metal bound to the protein varies depending on the apiary location and the sampling time. This is clear when comparing the profiles of honeybees from apiary M in April and May: zinc is predominantly present in the Mt peak in April, while copper is mostly present in this peak in May with zinc present in fractions 24–29, presumably bound to LMM molecules (Supplementary Figure S2). There is no information available in the literature regarding the metal specificity of honeybee Mt and this study provides the first evidence of its ability to bind either zinc or copper.

## 4. Conclusions

The environment, particularly agricultural practices and crop types, clearly affects the metabolism of proteins and trace elements in honeybees. Scarcity of wild flora or an altered bloom period due to climate change has a detrimental effect on nutrient availability, forcing honeybees to digest vitellogenin and other endogenous proteins to obtain free amino acids that can be used as metabolic substrates. Moreover, agricultural practices and the release of pesticides such as copper sulphate into the environment can alter trace element homeostasis. Therefore, agricultural policies that sustain pollinator-friendly ecosystems and practices must be adopted.

In the context of accelerating environmental and climate change, it is of critical importance that we improve our understanding of the intricate interrelationships between honeybees, their ecological context, and apicultural practices. Despite substantial progress, many fundamental research questions remain unanswered. A primary challenge is the implementation of robust field-based methodologies for assessing honeybee health and nutritional status of the colonies. The results of this research provide further evidence in this regard.

Finally, this study underscores the urgent need for integrative research frameworks that use multi-method approaches to address the multifaceted nature of this issue effectively. This holistic approach can provide a comprehensive view of the biochemical responses to environmental changes, pathogens, and feed availability that impact nutritional status and health of honeybees. Understanding these interactions will enable researchers to identify new biomarkers for early stress detection and improve hive management with a focus on honeybee resilience and health.

## Data Availability

The original contributions presented in this study are included in the Supplementary Material. Further inquiries can be directed to the corresponding author.

## Supplementary Materials

The following supporting information can be downloaded at: https://www.mdpi.com/article/10.3390/ani15182660/s1, Figure S1: Electrophoretic profiles and pherograms of fractions 11 obtained after SEC chromatography; Figure S2: Chromatographic profiles of zinc and copper after size exclusion chromatography (SEC) of extracts of honeybees; Table S1: Identification of the most abundant proteins present in the LMM bands (<14>10 kDa); Dataset S1: Metals chromatographies, Dataset S2: Proteins Chromatographies.
