# Supplementary material for: An Innovative Multi-Omics Approach Reveals the Interactions Between Honeybees and Their Environment
Source: Animals (Basel). 2025 Sep 11;15(18):2660. doi: 10.3390/ani15182660 (PMC12466506; doi:10.3390/ani15182660)
Supplement: Supplementary file 1 [file animals-15-02660-s001.zip › Supplementary Table and Figures.pdf]

Supplementary Materials

An Innovative Multi-Omics Approach Reveals the Interactions Between Honeybees and Their Environment

Cecilia Rudelli [1,*], Elisa Bellei [2], Giulia Andreani [1] and Gloria Isani [1]

[1] Department of Veterinary Medical Sciences, University of Bologna, 40064 Bologna, Italy
[2] Proteomic Laboratory, Department of Surgery, Medicine, Dentistry and Morphological Sciences, University of Modena and Reggio Emilia, 41125 Modena, Italy
* Correspondence: cecilia.rudelli2@unibo.it

Supplementary Table S1. Identification of the most abundant proteins present in the LMM bands (<14>10 kDa).

Protein name[(a)]	Organism	Score[(b)]	Cov.[(c)]	Match[(d)]	Seq.[(e)]	emPAI[(f)]	Gene name[(g)]
Vitellogenin (fragment)	*Apis mellifera*	943	75%	45	11	30.37	Vg
Vitellogenin (fragment)	*Apis mellifera*	941	82%	45	12	36.39	Vg
OBP14 precursor	*Apis mellifera*	6995	80%	283	18	18351.9	Obp14
OBP13	*Apis mellifera*	2895	68%	97	14	1822.97	Obp13
OBP21	*Apis mellifera*	1755	60%	51	10	48.63	Obp21
OBP18 precursor	*Apis mellifera*	1210	54%	36	10	20.49	Obp18
OBP3	*Apis mellifera*	1082	44%	29	7	5.11	Obp3
Chymotrypsin inhibitor	*Apis mellifera*	754	96%	26	4	63.45	LOC725380
Profilin	*Apis mellifera*	533	65%	16	5	4.29	LOC726426
Uncharacterized protein	*Apis mellifera*	476	52%	22	5	5.08	LOC102656354
Histone H4	*Apis mellifera*	336	38%	15	4	4.14	LOC102655073
Histone H2B	*Apis mellifera*	165	34%	10	5	4.50	LOC724869
Histone H2A	*Apis mellifera*	102	42%	12	5	4.73	LOC724678

[(a)]Recommended protein name from the UniProt database; [(b)]Score obtained with MASCOT MS/MS ions search; [(c)]Sequence coverage (percentage of amino acids sequenced for the detected protein); [(d)]Number of significant peptides matching the identified protein; [(e)]Number of significant protein sequences; [(f)]Exponentially modified protein abundance index; [(g)]Name of the gene that code for the protein sequence (UniProt database). OBP, odorant binding proteins.

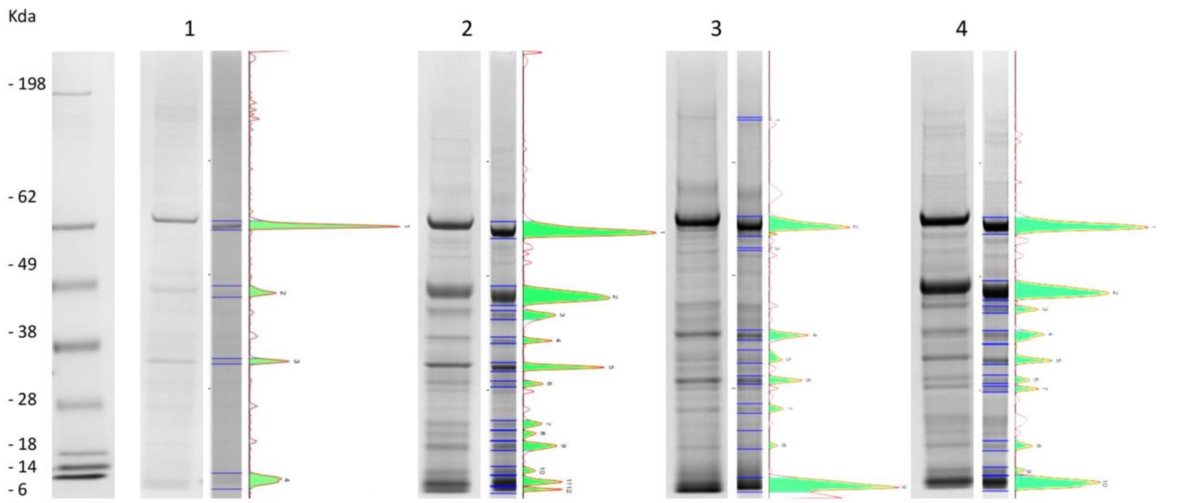

Supplementary Figure S1. Electrophoretic profiles and pherograms of fractions 11 obtained after SEC chromatography. Lane 1: apiary A in May; lane 2: apiary A in November; lane 3: apiary M in May; lane 4: apiary M in November. The molecular masses of the standard proteins are reported on the left.

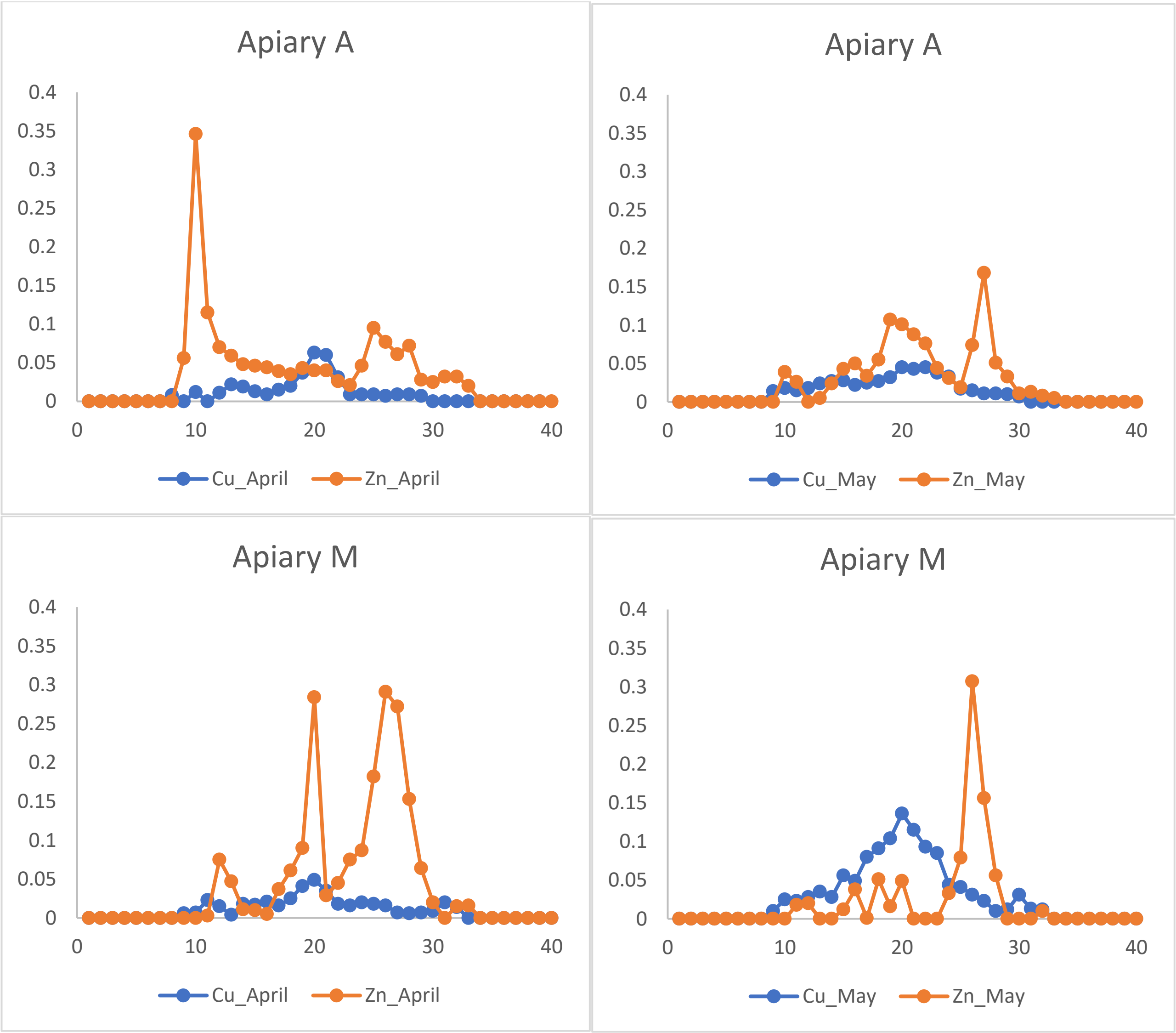

Supplementary Figure S2. Chromatographic profiles of zinc and copper after size exclusion chromatography (SEC) of extracts of honeybees sampled from apiaries A and M in April and in May. The concentration is expressed as µg/mL.
